# Interaction of Interleukin-17A with a Th2 Response in a Mouse Model of Allergic Airway Inflammation

**DOI:** 10.3390/cells12131774

**Published:** 2023-07-04

**Authors:** Karin Peters, Stefanie Ernst, Marcus Peters

**Affiliations:** 1 Department of Molecular Immunology, Ruhr-University Bochum, D-44780 Bochum, Germany; 2 Department of Experimental Pneumology, Ruhr-University Bochum, D-44780 Bochum, Germany

**Keywords:** Interleukin-17, Interleukin-13, asthma, mouse model, endotypes

## Abstract

Background: A total of 262 million people worldwide suffer from asthma and 461000 people died from it in 2019. Asthma is a disease with different endotypes defined by the granulocytes found in the asthmatic lung. In allergic asthma, the eosinophilic endotype is present, driven by a TH2 response. A TH17 immune response leads to the neutrophil endotype. This often causes uncontrolled asthma and is triggered by pollutants, microbes, and oxidative stress. It has been described that a significant number of patients with eosinophilic asthma develop mixed granulocytic asthma over time. The severity of asthma in the mixed endotype is related to the proportion of neutrophils in the lungs. Purpose: In this report, we address the question of how a TH2 response interacts with IL-17A in allergic asthma. Methods: To this end, we used a mouse model to induce allergic asthma followed by an aerosol challenge with ovalbumin. To investigate the role of IL-17A, we administered IL-17A intranasally during the challenge phase. Results: IL-17A alone did not elicit an immune response, whereas in combination with allergic asthma, it resulted in a shift of the asthmatic endotype from eosinophilic to neutrophilic. TGFβ1 was increased in these lungs compared to asthmatic lungs without IL-17A, as was the expression of the IL-17A receptor subunits IL-17RA and IL-17RC. In cultures with human cells, we also found that IL-17A increased the expression of its receptors only in combination with IL-13. We also found this effect for IL-8, which attracts neutrophils in humans. Conclusions: The TH2 response increased the sensitivity to IL-17A in a mouse asthma model as well as in human cell lines.

## 1. Introduction

Asthma is characterized by coughing, wheezing, chest tightness, and shortness of breath [1]. There is a narrowing of the airways due to the thickening of airway muscles, inflammatory infiltrates in the mucosa of the airways, and increased mucus production. During an asthma attack, the airway muscles contract and narrow the airways even further, causing life-threatening airflow limitation. There are different endotypes in asthma. These are characterized by the predominant granulocyte population. Eosinophilic asthma is thought to be based on an allergen specific immune response. A TH2 response is induced by repeated allergen contact. TH2 cells secrete IL-4 that stimulates B cells to produce allergen-specific IgE. This binds to IgE receptors on mast cells. Cross-linking of these receptors activates the mast cell and releases histamine, prostaglandins, and leukotrienes. These substances trigger a type 1 allergic response [2]. TH2 cells also secrete IL-13 and IL-5. IL-13 enhances airway hyperreactivity, while IL-5 attracts eosinophils to the lungs. Eosinophil granulocytes proliferate under the influence of IL-5 and, when activated, release their toxic granules, which include major basic proteins (MBP), cysteinyl leukotrienes, peroxidases, and reactive oxygen species (ROS), which further damage lung tissue [2,3]. In contrast to eosinophilic asthma, neutrophilic asthma is thought to be induced by the activity of TH17 cells. Bacteria and viruses, as well as particulate matter, can induce this immune response in the lungs. TH17 cells secrete Interleukin-17A. This binds to the IL-17 receptor complex on structural cells in the lung. It is a heterotrimer consisting of two subunits of IL17RA and one subunit of IL-17RC. IL-17A can bind to this complex as a homodimer or as a heterodimer with IL17F. In humans, IL-17A indirectly recruits neutrophils to the lung by stimulating the secretion of IL-8 in structural cells. A powerful tool of activated neutrophils is their ability to form neutrophil extracellular traps (NETs). These are web-like structures rich in host DNA and they contain modified histone proteins and granule proteins such as neutrophil elastase (NE) and myeloperoxidase (MPO) [4]. Initially discovered for their role in bacterial killing [5], NETs are now thought to contribute to the pathophysiology of various infectious or noninfectious diseases [6]. Interestingly, NETs can be formed in the lungs upon infection with respiratory viruses. Moreover, they can promote lung damage, thrombosis, and fibrosis. Unlike eosinophilic asthma, neutrophilic asthma is often not controlled by corticosteroids. Furthermore, according to [7], IL-17A increases the AHR after methacholine stimulation in humans and is associated with airway remodeling. In the context of airway remodeling, matrix metallopeptidase 9 (MMP9), a collagenase secreted by neutrophil granulocytes, is also reported to be increased in expression, which degrades the extracellular matrix [8]. IL-17A enhances epithelial secretion of mucus in mouse models and human cell models through increased expression of mucin 5AC (MUC5AC). In addition, IL-17A increases bronchial smooth muscle cell proliferation and migration and αSMA expression in fibroblasts [7]. Furthermore, IL-17A may enhance subepithelial fibrosis through increased expression of collagen and TGFβ in epithelial cells, which thereby adopt a mesenchymal phenotype as part of an epithelial–mesenchymal transition (EMT) [9]. Moreover, TGFβ correlates with increased TH17 activity in patients with severe asthma and is essential for IL-17A production [10].

From the literature, there are several indications that TH17 response can be observed in allergic asthma and exacerbate the disease burden of asthmatics. For example, Kerzel showed that there is a negative significant correlation between the frequency of TH17 cells and the level of asthma control in children with atopic asthma [11]. Nieminen et al. were able to show similar results in a study of sublingual immunotherapy in children [12]. In this study, allergen induced IL-17 mRNA was significantly increased in children with an elevated combined symptom medication score after two years in sublingual therapy. The same effect was shown in allergic asthmatic adults [13]; the percentages of TH17 cells as well as plasma concentrations of IL-17A and IL-22 tended to increase the severity of the disease. Cosmi et al. reviewed that TH2/TH17 dual positive cells can originate from TH2 or from TH17 cells due to their high plasticity and produce both IL-4 and IL-17 circulating in patients with bronchial asthma [14]. Moreover, Irvin et al. showed that TH2/TH17 cells from BALs of asthma patients where resistant to dexamethasone-induced cell death. In a T cell transfer model of atopic asthma in mice, IL-17 producing TH2 cells exacerbated asthma more strongly than conventional TH2 or TH17 cells alone [15]. 

In this study, we investigated the interaction of IL-17A with an allergic TH2 response in a mouse model of asthma. Our purpose was to figure out how IL-17A interacts with a TH2 response. Therefore, we induced allergic asthma with OVA, challenged mice several times, and applied IL-17A intranasally during provocation. We found that IL-17A does not influence the TH2 response, but it induced neutrophilia only when allergic asthma was induced. In this case, TGFβ1 expression was upregulated in the airways, as well as the expression of IL-17 receptor subunits RA and RC. IL-17A given to untreated mice could not induce neutrophilia, elevated TGFβ1 expression, or the upregulation of IL-17A receptors, but it could induce hypertrophy of the airway smooth muscles. In in vitro experiments with human cells, IL-17A in combination with IL-13 could upregulate the expression of IL-17RA and, subsequently, higher levels of IL-8 were induced.

## 2. Material and Methods

### 2.1. Animals

Female C57Bl/6, aged 6 weeks, were used. Mice were purchased from Janvier or Charles River and acclimated for two weeks to the animal facility before the experiments started. Mice had access to food and water ad libitum. All experimental procedures were approved by the animal ethics committee at Landesamt für Natur, Umwelt und Verbraucherschutz, Nordrhein-Westfalen, Germany (84-02.04.2013.A138).

To sensitize mice to the antigen OVA, an OVA/aluminum hydroxide (Alum) mixture was prepared. OVA Grade V (Sigma-Aldrich, Taufkirchen Germany) was dissolved at 5 mg/mL in PBS and sterile filtered. A total of 20 μL of the sterile OVA solution was incubated together with 630 μL of sterile PBS and 350 μL of aluminum hydroxide (ImjectAlum, Fisher Scientific, Schwerte, Germany) for 10 min on a roller shaker at RT. Each mouse was injected intraperitoneally with 200 μL OVA/Alum twice, each injection five days apart.

**Figure 1:**
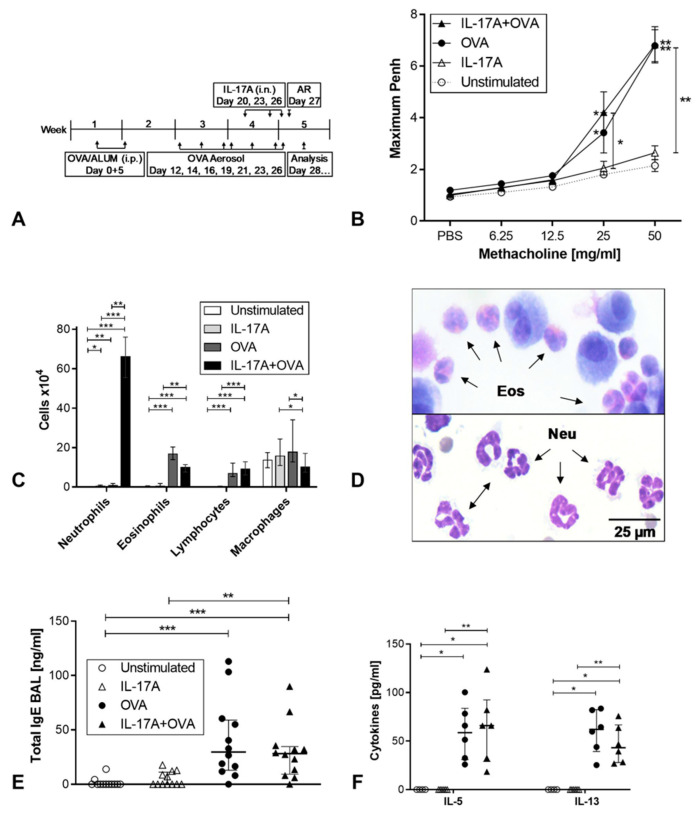
Allergic inflammation in the airways of mice. (**A**): Treatment of Balb/c mice, Group1: unstimulated *n* = 8, Group 2: IL-17A application intranasally (i.n.) *n* = 12, Group 3: Ovalbumin (OVA)-asthma *n* = 12, Group 4: OVA-Asthma + IL-17A i.n. *n* = 12 (**B**): Airway hyperreactivity measured by whole body plethysmography. (**C**): Cell counts in bronchioalveolar lavage (BAL). (**D**): Cells in BAL cytospins, upper panel: OVA group, arrows indicate eosinophils, lower panel: IL-17A + OVA group, arrows indicate neutrophils. (**E**): Total IgE in BAL fluid. (**F**): Restimulation of spleen cells with OVA: IL-5 in supernatants after 48 h (**left**), IL-13 in supernatants after 48 h (**right**). Statistical differences were calculated using a one-way ANOVA Kruskal–Wallis Test; for differences to control groups, Dunn’s post-test was used. * *p* < 0.05; ** *p* < 0.01; *** *p* < 0.001.

Induction of inflammation in the lungs began on day twelve by provocation with OVA aerosol (aerosol challenge). For this purpose, sensitized mice were transferred to a Plexiglas chamber. An amount of 5 mL of a 1% OVA solution (*w*/*v*) was nebulized with the PariBoy Turbo nebulizer (type 038) within 20 min on days 12, 14, 16, 19, 21, 23, and 26. The scheme of experimental procedures is shown in Figure 1A. To apply rm IL-17A intranasally (IL-17A: ImmunoTools, Friesoythe, Germany) on days 20, 23, and 26, animals were anesthetized with a mixture of ketamine and xylazine followed by intranasal instillation of 2 µg IL-17A in 50 µL PBS. One day after the last provocation, airway hyperreactivity was analyzed using whole body plethysmography (see below). The next day (28), mice were sacrificed for analysis. 

In addition, a long-term provocation protocol was used. Here, OVA sensitization and provocation were performed as described above, but the number of provocations was increased by further administration of OVA aerosol on days 28, 30, 33, 35, 34, and 40. In addition to the administration of IL-17A on days 20, 23, and 27, it was administered on days 30, 34, 37, and 40 (see also Supplemental Figure S5A).

### 2.2. Whole Body Plethysmography

A whole-body plethysmograph (MAX II 2270 BUXCO Bias Flow Regulator und BUXCO Aerosol Nebulizer Control 10, Buxco Electronics, Wilmington, NC, USA) was used to analyze the respiratory hyperreactivity of mice to methacholine. For this purpose, methacholine was first dissolved in PBS and stored on ice. The mice were each placed in a chamber of the plethysmograph, which had been previously calibrated according to the manufacturer’s instructions. After a few minutes in the chamber, lung function measurements began with the establishment of the baseline PenH value by nebulizing 200 μL of PBS with the nebulizer and steadily delivering it to the mice over 1.5 min. Subsequently, aerosols of 200 μL methacholine solution (6.25 mg/mL, 12.5 mg/mL, 25 mg/mL, and 50 mg/mL) each were steadily introduced into the chambers in ascending concentrations for 1.5 min each. During the measurement, PenH and vital signs were monitored so that intervention could be made, and the measurement stopped if necessary.

### 2.3. In Vitro Cytokine Production of Mouse Lymphocytes

Spleens were harvested two days after the last aerosol challenge. Single cell suspensions were prepared from spleens by mechanical disruption or from lungs by digestion with collagenase type III from *Clostridium histolyticum* (0.5 mg/mL, Sigma -Aldrich, Taufkirchen Germany). Erythrocytes were lysed by hypotonic shock. Lymphocytes were then cultured at a concentration of 10^7^/mL in complete tissue culture medium (RPMI 1640 with 10% fetal calf serum (FCS), 2 mM L-glutamine, 100 U/mL penicillin, and 100 µg/mL streptomycin, all from Biochrom, Berlin, Germany). Cells were restimulated with OVA. After 48 h of culture, supernatants were taken and stored at −80 °C until analysis. 

### 2.4. Preparation of Murine Lungs

The right lobe of the lung was ligated so that the left lung was filled with 4% paraformaldehyde (PFA) and harvested. The left lungs were stored at 4 °C for 72 h. From this, paraffin sections were prepared. The right lung lobes were removed and frozen dry at −80 °C. They were used for RNA extraction.

### 2.5. ELISA

For quantification of mIL5, mIL13, mTGFβ1, mIgE, hIL-8, and hTGFβ1, the corresponding ELISA kits were used. The procedure was performed according to the manufacturer’s instructions. According to the experience of the Experimental Pneumology Bochum, no cytokines were detected in the analysis of the supernatant of OVA spleen cell stimulation of naïve C57Bl/6 mice. CXCL1 was detected in BAL fluid by using a KC/CXCL1 Mouse ELISA Kit from Thermo Fisher Scientific (#EMCXCL1).

### 2.6. Bronchoalveolar Lavage (BAL)

Three days after the last OVA challenge, lungs were lavaged via a tracheal cannula with 2 × 1 mL PBS, and leukocytes in the lavage fluid were counted. After centrifugation, BAL fluid was frozen for further analysis. Cytospin slides of BAL cells were stained with a quick staining procedure (HAEME-Schnellfärbung, Labor + Technik Eberhard Lehmann, Berlin, Germany), according to the manufacturer’s instructions. The percentages of eosinophils, lymphocytes, and macrophages in BAL samples were determined by light microscopy. At least 300 cells per sample were differentiated by a blinded investigator. The concentration of total protein in BALF supernatant was detected by a conventional Bradford assay by using bovine serum albumin as a standard protein. 

### 2.7. Histology

Lungs were fixed by inflation with 1mL of 4% buffered PFA via a tracheal tube. Further fixation was carried out by incubation of the lungs in the same fixation medium for 24 h. Afterwards, lungs were embedded in paraffin and sliced to 5 µM sections with a microtome. After deparaffinization, slices were stained for goblet cells with Periodic acid Schiff (PAS) staining and fibrotic tissue with Masson Goldner’s Trichrome staining. Stained slices were analyzed using a light microscope (Olympus BX40, Olympus; Hamburg, Germany). Photographs were taken using a digital microscope camera (moticam 1 SP, Motic Deutschland GmbH, Wetzlar, Germany). A digital image analysis was performed using ImageJ (National Institutes of Health). In order to analyze fibrosis, the thickness of the basement membrane was measured. Therefore, five images of each lung slice were taken, and the thickness of the green stained collagen layer was determined within five samples from each image using the “straight segmented line” tool (ImageJ; National Institutes of Health). In order to quantify goblet cell hyperplasia after PAS staining, five images of each lung slice were taken and mucus producing cells in PAS-stained tissue were counted, length of the airway epithelium was measured using the “straight segmented line” tool, and the number of positive cells per µm was calculated. 

### 2.8. RNA Isolation and cDNA Synthesis

RNA isolation was performed by chloroform–phenol extraction. Lung tissue stored at −80 °C was taken up in 250 μL Trizol (Thermo Fisher Scientific, Waltham, MA) and processed in a mechanic homogenizer. Together with 50 μL chloroform, the suspension was shaken for 15 s and incubated at RT for 3 min. The solution was then centrifuged (12000× *g*, 15 min, 4 °C). The aqueous supernatant was transferred to a reaction tube and mixed with 125 μL isopropanol. After the solution was incubated for 10 min at RT, it was centrifuged at 12000× *g* (10 min, 4 °C). The supernatant was discarded and 250 μL of 75% ethanol solution (*v*/*v*) was added. After a brief vortexing, the suspension was centrifuged again (7500× *g*, 5 min, 4 °C) and the supernatant discarded. The sediment was then dried for 5–15 min at RT before resolubilization with 30 μL RNase-free water and frozen at −20 °C.

The RNA concentration was determined photometrically. Subsequently, cDNA synthesis was performed using the High-Capacity cDNA Reverse Transcription Kit (Thermo Fischer, Scientific, Waltham, MA, USA), which was used according to the manufacturer’s instructions. Subsequently, the 20 μL cDNA solution was diluted with 50 μL RNase-free water.

### 2.9. Real Time PCR

The GoTaq^®^ qPCR Kit (Promega) was used to quantify specific RNA transcripts. Therefore, it was mixed on ice in 48-depot PCR plates with a total volume of 20 μL. They each consisted of 10 μL 2× Master Mix, 0.2 μL CXR Reference Dye, 1 μL of the specific primer mix (10 pmol forward and 10 pmol reverse primer), 4 μL of the diluted cDNA solution, and 4.8 μL RNase-free water. After a brief centrifugation of the plate, analysis was performed in the StepOne™ Real-Time PCR System (Applied Biosystem). Amplification of the specific transcripts was performed by a 10 min denaturation step at 95 °C followed by 40 PCR cycles. Each cycle included a 15-s denaturation phase at 95 °C, and an attachment and synthesis phase at 60 °C for 1 min. The relative concentrations of RNA transcripts were calculated according to the ΔΔCT method.


**Protein**

**Forward Primer**

**Reverse Primer**
mIl17RAAGTTCCAGTTTCTGTCCATGC TGGATTTGTGGTTTGGGTCmIl17RCGCAGAGCCTGAAGAAGCTG CCCAAGACTAGCCTCGAAACmRplp0CGTCCTCGTTGGAGTGACAT TAGTTGGACTTCCAGGTCGCmMUC5ACCCATGCAGAGTCCTCAGAACAA TTACTGGAAAGGCCCAAGCAmTGFβ1CTCCCGTGGCTTCTAGTGC GCCTTAGTTTGGACAGGATCTGhIL-17RAGCTTCACCCTGTGGAACGAAT TATGTGGTGCATGTGCTCAAAhIL-17RCGATGGTGACAACGTGCATCTG CAAGGTAATGATCTGCGGTCChIL-8CTTGGCAGCCTTCCTGATTT TTCTTTAGCACTCCTTGGCAAAAhRPLP0CTGGAAGTCCAACTACTTCCT CATCATGGTGTTCTTGCCCAThTGFβ1GGCGTGCTAATGGTGGAAAA TGTGTGTACTCTGCTTGAACTTGTCA

### 2.10. Immunofluorescence Staining and Detection

Tissue sections were deparaffinized and rehydrated, followed by antigen retrieval with 0.8% SDS for 10 min. Blocking and permeabilization were performed by incubating sections in 10% fetal bovine serum and 0.5% TritonX-100 for 30 min. Sections were incubated for 90 min with mouse anti-αSMA primary antibody 1:400 in PBS with 1% FCS (A2547; Sigma-Aldrich, St. Louis, MO, USA) and after washing for 60 min with secondary goat antibody conjugated with AF488 1:1000 in PBS (ab150141; Abcam, Cambridge, UK). Cell nuclei were counterstained with Roti-Mount FluorCare DAPI (Roth, Karlsruhe, Germany). All steps were performed at RT.

### 2.11. Cell Culture

Fetal lung fibroblasts (MRC5) were obtained from ATCC at population doubling 28. Cells were passaged according to the ATCC protocol in MEM-Eagle containing 10% FCS, 2 mM L-glutamine, 100 U/mL penicillin, and 100 µg/mL streptomycin, all from Biochrom, Berlin, Germany. The carcinogenic lung epithelial cell line (A549) was cultured in DMEM, 10% FCS, 2 mM L-glutamine, 100 U/mL penicillin, 100 µg/mL streptomycin. Cells were stimulated with recombinant IL-13 and IL-17A (Immuno Tools). Per well of a 24 well plate, 35000 cells were inoculated in a volume of 500 µL. The cells were incubated for 24 h under the given conditions. This was followed by stimulation with IL-13 and/or IL-17A at a concentration of 10 ng/mL and/or 100 ng/mL in DPBS or TGFβ1 (Immuno Tools; 10 ng/mL). As a negative control, DPBS was added to the cell suspension. The plates were incubated for 24 h at 37 °C and then centrifuged (1000 rpm, 10 min). The cell supernatants were decanted into 1.5 mL reaction tubes and stored at −80 °C. The cytokine IL-8 was quantified by ELISA. Cells were washed with 1 mL DPBS/well and RNA was finally extracted with TRI reagent (Merck, Sigma Aldrich, Taufkirchen, Germany).

### 2.12. Statistical Analysis

Data were analyzed by the Kruskal–Wallis test. If a significant difference was found, treated groups were compared with the control group by using the Dunn’s post-test. For analysis of correlation, the non-parametric Spearman’s test was applied. GraphPad Prism Software (Version 8.0) was used for analysis. Values of *p* < 0.05 were considered statistically significant.

## 3. Results

### 3.1. Impact of IL-17A on Allergic Inflammation in a Mouse Model of Asthma

To evaluate the role of IL-17A in allergic inflammation and asthma, we chose a mouse model using the chicken ovalbumin as the model allergen (Figure 1A). Mice were sensitized by twice intraperitoneal administration of ALUM-absorbed OVA. One week after the second sensitization, we started the provocation with OVA aerosol. In total, we provoked seven times, three times a week. After the fourth provocation, we started to give IL-17A intranasally on three days (20, 23, 26). Finally, one day after the last provocation, airway hyperreactivity was measured before mice were sacrificed for analysis on day 28. Four groups were compared: one unstimulated control group, which was left completely untreated, the IL-17A group, which was solely treated with IL-17A intranasally, the OVA group which was sensitized and provoked, and the IL-17A + OVA group which was sensitized, provoked, and received IL-17A. One day after the last provocation and IL-17A application, airway hyperreactivity was analyzed by whole body plethysmography upon stimulation with raising concentrations of methacholine (Figure 1B). The maximum PenH revealed an airway hyperreactivity in the two asthma groups compared to the two control groups without asthma, which was statistically significant for both asthma groups. Between the two asthma groups, differences were only marginal and not statistically significant. The composition of cell types in the bronchioalveolar lavage (BAL) showed that the application of IL-17A alone did not induce a neutrophilia in the lung. In combination with allergic asthma, IL-17A induced a strong neutrophilia (Figure 1C,D). In contrast, in the OVA asthma group, airway eosinophilia, but no neutrophilia was observed. Additionally, IL-17A reduced the number of eosinophils, but this was not significant. In Figure 1D, a cytospin showing airway eosinophilia from one mouse in the OVA group (upper panel) in contrast to neutrophilia of a mouse of the IL-17A + OVA group in the lower panel is presented. The amount of total IgE measured in the BAL fluid was significantly increased in the OVA and IL-17A + OVA group compared to non-asthma control groups, but no difference could be observed between the asthma groups (Figure 1E). Spleen cells were cultured and restimulated with OVA. IL-5 and IL-13 secretion was analyzed by ELISA, and again, the two TH2 cytokines were elevated in asthma groups, but the amounts of secreted cytokines were similar in the asthma groups. In contrast, by measuring CXCL1 as a chemoattractant for neutrophilic granulocytes in the supernatant of BALF, we found a significantly increased concentration of this chemokine in the lungs of mice treated with IL-17A + OVA compared with mice treated with OVA only (Supplemental Figure S1A). These results show that IL-17A does not influence the TH2 response in atopic asthma in mice, but it induces an airway neutrophilia in the context of allergic asthma, while IL-17A alone is not sufficient for this effect. Surprisingly, there does not appear to be an exacerbation of disease associated with the increased neutrophilia, as there is no evidence of clinical deterioration of the disease, i.e., no weight loss, no respiratory symptoms, and no increased total protein content in the BALF as an indicator of increased tissue destruction (Supplemental Figure S1B).

### 3.2. Analysis of Airway Remodeling

**Figure 2:**
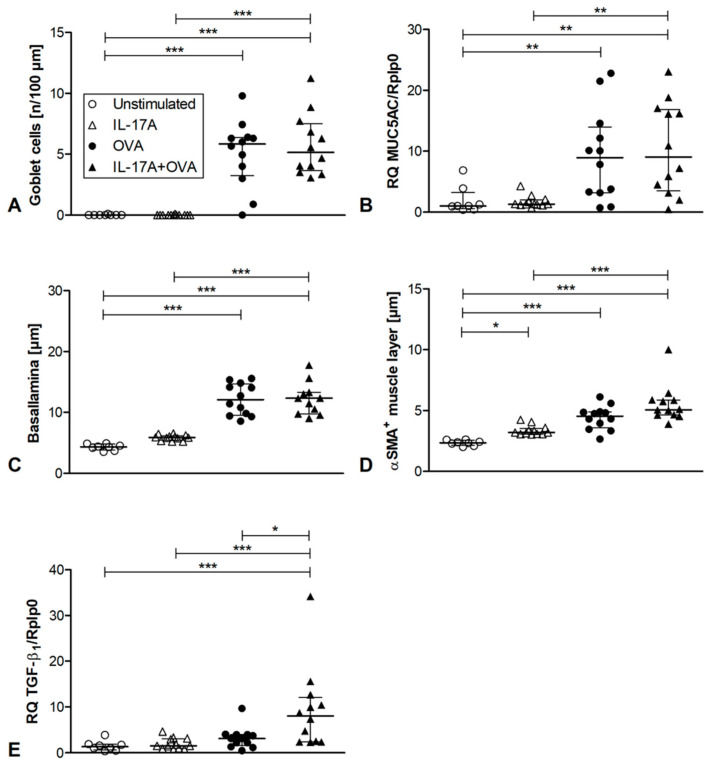
Airway remodeling and TGFβ1 expression. (**A**): Goblet cells in proximal airway epithelium, cells, per 100 µm of Alician blue PAS-stained lung sections. (**B**): Expression of MUC5AC in whole lung tissue compared to the housekeeping gene RPLP0 with ΔΔCT-method (**C**): Thickness of the basal lamina in proximal airways measured after sirius red staining, one datapoint reflects 25 measurements of 5 bronchioles from one mouse. (**D**): Thickness of smooth muscle layer of proximal airways, analyzed upon immunofluorescence staining of αSMA positive cells, one datapoint reflects 25 measurements of 5 bronchioles from one mouse. (**E**): Expression of TGFβ1 in whole lung tissue compared to the housekeeping gene RPLP0 with ΔΔCT-method unstimulated *n* = 8, IL-17A *n* = 12, OVA *n* = 12, IL-17A + OVA *n* = 12. Statistical differences were calculated using a one-way ANOVA Kruskal–Wallis Test; for differences to control groups, Dunn‘s post-test was used. * *p* < 0.05; ** *p* < 0.01; *** *p* < 0.001.

Next, we looked for airway remodeling parameters. In the airway epithelium, mucus producing goblet cells occur in atopic asthma. By Alician Blue PAS staining, we saw increasing numbers of goblet cells in the asthma groups compared to non-asthma controls, independent of IL-17A administration (Figure 2A and Figure S2). The expression of MUC5AC had the same pattern, which was expected as the mucus is produced by goblet cells (Figure 2B). Thickness of the basal lamina of the airway epithelium was specified by sirius red staining (Figure 2C and Figure S3). Again, asthma groups showed thickened basal lamina compared to non-asthma controls. There is a trend for increased thickness by administration of IL-17A, but this effect is not statistically significant. By immunofluorescence staining against αSMA, we could analyze the thickness of the smooth muscle layer of the proximal airways. Here, IL-17A administration alone increased the thickness compared to unstimulated controls. By inducing asthma, the thickness further increased (Figure 2D and Figure S4). TGFβ1 is a profibrotic factor. Moreover, depending on the conditions, it can regulate the immune response. For instance, in combination with IL-6, it can upregulate TH17 cells, whereas without IL-6, it can activate regulatory T cells. In whole lung tissue, it was upregulated in the IL-17A + OVA asthma group compared to all other groups (Figure 2E). Although the combination of IL-17A and OVA resulted in a significant increase in TGFβ1, no enhanced remodeling was observed. To test whether this observation was due to a short challenge period, we repeated the experiment and increased the number of challenges. However, even with six additional challenges, we did not observe a significant worsening of remodeling (Supplemental Figure S5D,E).

### 3.3. Investigation of the IL-17 Receptor Expression In Vivo and In Vitro

**Figure 3:**
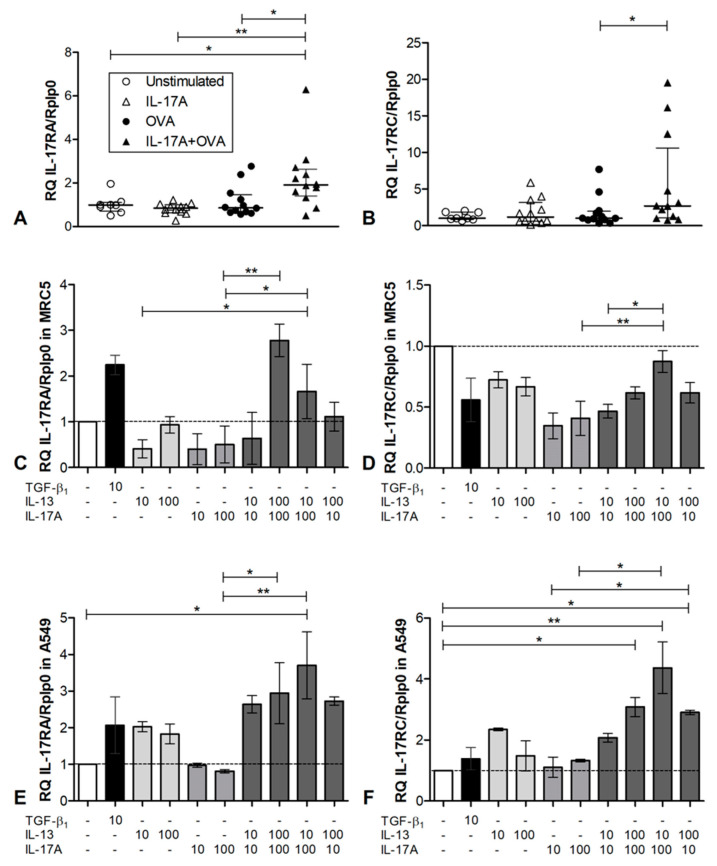
In vitro expression of IL-17 receptor subunits. (**A**): Expression of IL-17RA in whole lung tissue compared to the housekeeping gene RPLP0 with ΔΔCT-method. (**B**): Expression of IL-17RC in whole lung tissue compared to the housekeeping gene RPLP0 with ΔΔCT-method; unstimulated *n* = 8, IL-17A *n* = 12, OVA *n* = 12, IL-17A + OVA *n* = 12. Statistical differences were calculated using a one-way ANOVA Kruskal–Wallis Test; for differences to control groups, Dunn‘s post-test was used. * *p* < 0.05; ** *p* < 0.01. (**C**): IL-17RA expression in MRC5 human fetal lung fibroblasts upon stimulation with cytokines, compared to the housekeeping gene RPLP0 using ΔΔCT-method *n* = 3. (**D**): IL-17RC expression in MRC5 human fetal lung fibroblasts upon stimulation with cytokines *n* = 3. (**E**): Expression of IL-17RA in A549 cells *n* = 3, 35000 A549 cells per well were seeded in a 24 well microtiter plate. After 24 h, cells were stimulated as indicated. After another 24 h, cells and supernatants were harvested for analysis. (**F**): Expression of IL-17RC in A549 cells.

To figure out why IL-17A only had an impact on neutrophilia in combination with atopic asthma, we analyzed the expression of the IL-17A receptor subunits A and C, which form a heterodimer to recognize IL-17A. We could observe an upregulation of IL-17RA in mouse whole lung tissue solely in the IL-17A + OVA group, which was statistically significant compared to the other groups (Figure 3A). Moreover, the receptor subunit C expression was statistically significant when upregulated in the IL-17A + OVA group compared to the OVA group (Figure 3B). 

To gain insights into the mechanism of the upregulation of the IL-17A receptor subunits, we performed cell culture experiments. We took the human fetal lung fibroblasts MRC5 and the human cancerous alveolar epithelial cell line A549 to stimulate them with IL-13 and IL-17A, solely, or in combination with two different concentrations. TGFβ1 was also tested alone. In MRC5 fibroblasts, TGFβ1 upregulated the IL-17RA expression two-fold. Application of IL-13 or IL-17A alone could not increase the expression of IL-17RA, but the combination of both heightened the expression, when the 100 ng/mL concentration of IL-17A was used (Figure 3C). In MRC5, no upregulation of IL-17RC could be observed. In A549 cells, TGFβ1 or IL-13 upregulated IL-17RA expression, while IL-17A did not show this effect. When IL-13 and IL-17A were administered simultaneously, IL-17RA expression was upregulated. In the combination of 10 ng/mL IL-13 and 100 ng/mL of IL-17A, this effect was statistically significant compared to the unstimulated control, while the 100 ng/mL concentration plus IL-13 in 10 or 100 ng/mL was significantly elevated compared to IL-17A in 100 ng/mL concentration alone (Figure 3D). IL17-RC could also be upregulated by co-stimulation with IL-13 and IL-17A, but not with IL-17A alone (Figure 3E). Here the low IL-13 concentration (10 ng/mL) in combination with the high IL-17A concentration (100 ng/mL) showed the strongest effect, but other combinations produced also significant upregulations. In a similar pattern, the IL-17RC subunit was regulated (Figure 3F). Taken together, the cell culture experiments show that IL-17A alone has no influence on its receptor subunit regulation, but together with the TH2 cytokine IL-13, IL-17RA and RC are upregulated and heighten the IL-17A sensitivity of the cells.

### 3.4. Investigation of IL-8 Expression by Stimulation with IL-17A and IL-13 of Human Cell Lines

**Figure 4:**
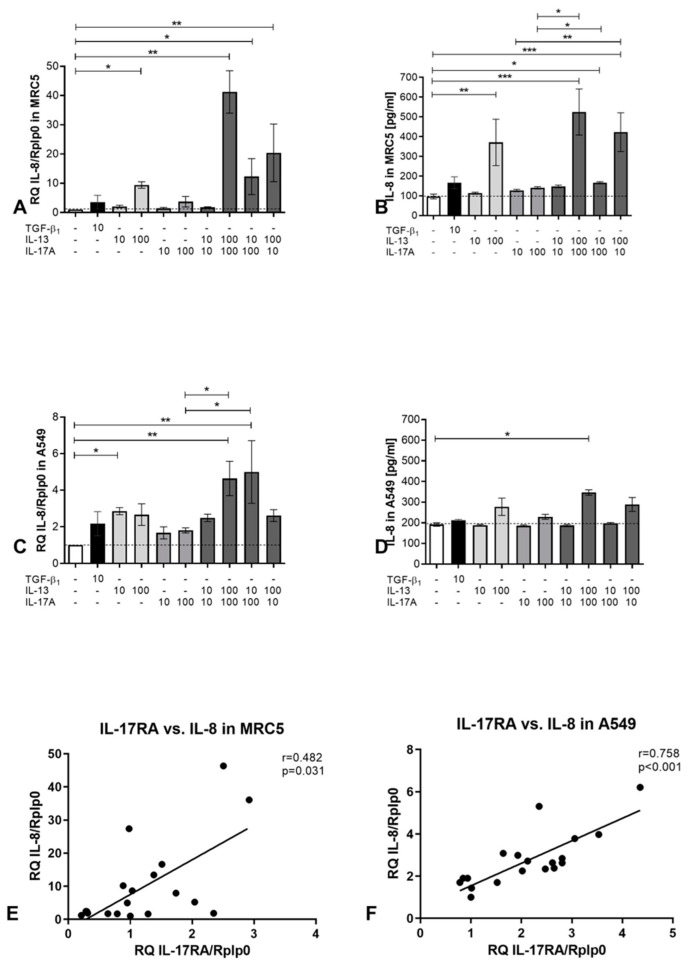
In vitro expression and secretion of IL-8 in response to cytokine stimulation. (**A**–**D**): 35000 MRC5 cells (**A** + **B**) or A549 cells (**C** + **D**) per well were seeded in a 24 well microtiter plate. After 24 h, cells were stimulated as indicated (ng/mL). After another 24 h, cells and supernatants were harvested for analysis. (**E**): Correlation of IL-17RA versus IL-8 expression in MRC5 cells. (**F**): Correlation of IL-17RA versus IL-8 expression in A549 cells. Statistical differences were calculated using a one-way ANOVA Kruskal–Wallis Test, for differences to control groups, Dunn‘s post-test was used. (*n* = 4), * *p* < 0.05; ** *p* < 0.01; *** *p* < 0.001.

Induction of the chemokine IL-8 is a main function of IL-17A. In a second step, IL-8 attracts neutrophils to the site of infection. For this reason, we measured the IL-8 expression and secretion of IL-8 of the MRC5 and A549 cells upon stimulation with IL-13 or/and IL-17A. In MRC5 cells, IL-8 was upregulated on the transcriptional level clearly by 100 ng/mL IL-13 and less with IL-17A 100 ng/mL. In combination, these two cytokines elevated the transcription of IL-8 in a synergistic way (Figure 4A). If one of these two factors were applied in the lower concentration (10 ng/mL), this effect was still visible, but to a lesser extent. By measuring the amount of IL-8 in the cell culture supernatants, IL-13 in the 100 ng/mL concentration induced the secretion of IL-8 into the supernatant. Further addition of 100 ng/mL Il-17A increased the amount in tendency but was not significant at this timepoint (Figure 4B). While MRC5 increased expression in high amounts, almost 40-fold when incubated with IL-13 and IL-17A in the high concentration (100 ng/mL), the reaction of A549 was lower, by a maximum of up to 4,5-fold (Figure 4C). An amount of 10 ng/mL of IL-13 induced a significant upregulation of IL-8 expression, while the combination of IL-13, independent of its concentration, combined with 100 ng/mL IL-17A induced a statistically significant upregulation compared to unstimulated as well as to 100 ng/mL IL-17A alone. The secretion of IL-8 by A549 cells showed only small differences (Figure 4D). Just the combination of 100 ng/mL IL-13 with 100 ng/mL IL-17A resulted in a significant secretion increase compared to the unstimulated control. The expression of IL-17RA and IL-8 shows a weak but significant correlation in MRC5 cells (Figure 4E), while this correlation is strong and highly significant in A549 cells (Figure 4F). This underlines the known dependence of IL-17A signaling and IL-8 production.

## 4. Discussion

The aim of our study was to investigate the influence of IL-17A on allergen-induced TH2-mediated allergic inflammation in the lung. In this context, the infiltration with inflammatory cells into the lung and the hyperreactivity of the airways were investigated. In addition, we focused on airway remodeling.

### 4.1. Influence of IL-17A on Th2-Mediated Inflammation in the OVA Mouse Model

In our study, wild-type mice were treated with recombinant IL-17A (i.n.) three times in an OVA asthma model to follow the influence of IL-17A on the development and progression of allergic asthma. Airway hyperreactivity was induced in allergic asthma by IL-13, a key TH2 cytokine. In this work, both asthma groups, OVA-only and IL-17A + OVA, had significantly increased AHR compared to non-asthma controls, but there was no difference in the asthma groups. It has been previously shown that AHR in murine asthma models is increased by additional stimulation with IL-17A [7,16,17]. In contrast to our experiments, in which we applied 2 µg IL-17A, Hall et al. showed that A/J mice had an increase in an IL-13-induced AHR only when 5 μg IL-17A was applied. Therefore, the concentration of IL-17A in our experiments was probably too low to see an effect on the AHR.

Inflammation of the airways was determined by differentiating cell types in the BALF using cytospins. The relative percentage of neutrophilic granulocytes in the IL-17A group was 3.3%, which in the OVA group was 2.4%, but in the IL-17A + OVA group was 68%. Thus, it can be concluded that a combination of IL-17A with a TH2 response causes a strong influx of neutrophils into the lung. It has been demonstrated by others that IL-17A application can induce neutrophilia in murine allergic asthma [7,10,17,18]. Neutralization of IL-17A in OVA asthma leads to a decrease in neutrophil granulocytes in the BAL [19]. However, the importance of neutrophil granulocytes for the pathogenesis of asthma remains unclear [20].

In summary, nasal administration of IL-17A significantly increased the proportion of neutrophil granulocytes in the airways. However, if a TH2 reaction occurred simultaneously in the lungs, the IL-17A-induced neutrophilia was strongly increased.

### 4.2. Influence of IL-17A on Airway Remodeling in the OVA Mouse Model

The characteristics of the pathophysiological remodeling of the bronchi were investigated here. First, the degree of fibrosis of the bronchial sub epithelium was verified by staining subepithelial collagen deposits. IL-17A thickened the collagen layer compared with control mice slightly but not significantly. The groups OVA and IL-17A + OVA also exhibited a thickened basement membrane compared with unstimulated or IL-17A treated mice, but this was independent of IL-17A. This contrasts with our previous data [21]. In this study, we observed an increasing thickness of the basal lamina with increasing amounts of IL-17A secreted by lung lymphocytes. One explanation might be that lung lymphocytes produce IL-17A steadily, while the IL-17A applied intranasally has a half-life, which is too short to influence the thickening of the collagen layer. 

TGFβ1 has been studied as another profibrotic mediator essential for TH17 cell differentiation and thus IL-17A production [10]. TGFβ1 is involved in airway remodeling by enhancing fibrosis and influencing bronchial muscle contraction. In addition, it is also secreted by neutrophil and eosinophil granulocytes [22]. In the experiments of this work, mRNA for TGFβ1 was significantly increased in the IL-17A + OVA group compared to the unstimulated controls and to the OVA group. Here, the relative gene expression of TGFβ1 was additively increased from 1.4 (IL-17A group) or 4.5 (OVA group) to 7.4 (IL-17A/OVA group). It is known that IL-17A enhances TGFβ1 signaling, and that the two cytokines can regulate each other by interacting through diverse signaling pathways. These include RhoA-, MAPKs-, and PI3K- (phosphoinositide 3-kinases) induced signaling cascades [10,23]. It is not yet clear whether the increased TGF production has any significance for the pathophysiological process.

Airway obstruction in asthma is associated with the production of viscous mucus secreted by goblet cells in the bronchial epithelium. The increased expression of MUC5AC in TH2 asthma was likely induced directly by the TH2 cytokine IL-13, which has been shown to increase MUC5AC in both human airway epithelial cells and mouse models [24,25]. There was no apparent influence of IL-17A.

In an OVA mouse model, it has been shown that airway smooth muscle cells cannot secrete IL-17A themselves but possess the IL-17RA/C receptor complex that enables them to bind IL-17A [26]. In the present work, the thickness of the bronchiolar muscle layer was examined based on the expression of αSMA. The smooth muscle layer in the groups IL-17A, OVA, and IL-17A + OVA was significantly thickened than in the unstimulated control group. In the IL-17A group, the bronchiolar muscle layer was on average 3.4 μm thick, in the OVA group 4.4 μm, and in the combined IL-17A/OVA response group 5.6 μm thick, suggesting an enhancing effect of IL-17A on thickening of the muscle layer. 

Overall, IL-17A was shown to have an enhancing effect on airway remodeling, as IL-17A-stimulated mice had a thickened basement membrane and muscle layer of bronchioles both proximally and distally. Allergic asthma to OVA also enhanced airway remodeling. Although the combination of IL-17A and OVA resulted in significantly increased TGFβ1, no increased remodeling was observed. This observation is somewhat surprising since neutrophilic granulocytes are thought to lead to tissue destruction with subsequent repair processes leading to enhanced fibrosis [27]. However, it is possible that under the particular circumstances in which neutrophils are attracted to the lungs in our study, the cells remain in a quiescent state [28]. In a resting state, neutrophils do not release toxic metabolites or perform netosis, however upon stimulation with, e.g., pathogen associated molecular patterns they become activated and progress into a primed neutrophil [28]. Future studies should address this question and investigate how lung pathology exacerbates once the infiltrating neutrophils become activated. This could be achieved by administering an additional stimulus such as LPS on days when granulocytes are already present in the lungs.

### 4.3. Mechanisms of Action of IL-17A in the OVA Mouse Model

IL-17A binds to its effector cells via a heterotrimeric receptor complex. The receptor complex consists of the IL-17RA/C subunits. In this work, we investigated whether subunit expression was enhanced in a combined IL-17A/TH2 response, which could explain the observed infiltration of neutrophilic granulocytes. The mRNA of both subunits was increased in a combined IL-17A/TH2 response, whereas administration of IL-17A alone was not sufficient to enhance expression of the receptor. Thus, the increased neutrophilic attraction observed in the IL-17A/OVA group can be explained by an enhanced expression of IL-17RA which is the subunit with the highest affinity for IL-17A. Several publications confirm this for the mouse model and humans. Moreover, it has been described that the affinity of IL-17RC to IL-17F is higher than to IL-17A [29,30]. Indeed, we found a significant increase in the concentration of CXCL1 in the BAL of mice treated with IL-17A + OVA compared with treatment with IL-17A alone. Thus, the Th2 milieu generated by OVA treatment appears to increase sensitivity to IL-17A. The increased expression in the IL-17 receptor seems to be an obvious explanation for this.

### 4.4. Significance of IL-17A for Human Lung Fibroblasts and Epithelial Cells

In the present work, we attempted to transfer the obtained results of murine asthma models to human cell culture systems. For this purpose, human pulmonary fibroblasts (MRC5) and epithelial cells (A549) were stimulated with IL-17A and the TH2-cytokine IL-13 for 24 h. The expression of IL-17 receptor subunits in this work showed that both IL-17RA but also IL-17RC were expressed by the human epithelial cells, so a functional receptor complex allowed signal transduction. In human fibroblasts, however, the IL-17RC subunit was not upregulated by adding IL-17A and/or IL-13. It remains questionable whether this was due to the stimulation time. IL-17A mediates the production of IL-8 via its receptor complex, which is mainly secreted by airway epithelial cells [7,31]. The fact is that IL-8 was synergistically increased by IL-17A not only in human epithelial cells but also in fibroblasts, at both gene and protein levels. In fact, compared with epithelial cells, it was significantly stronger. This indicates that IL-17A must have mediated signals to the fibroblasts. A positive correlation between IL-8 and IL-17RA was observed for the fibroblasts and for the epithelial cells. Further studies should include additional chemoattractants for neutrophil granulocytes such as leukotriene B4, anaphylatoxins C3a and C5a, the oncogene GROα (CXCL1), and the chemokine ENA75 (CXCL5). Interestingly, TGF-β1 stimulation could also induce IL-17RA expression with comparable level in MRC5 cells. Moreover, our mouse model showed that TGF-β1 expression was significantly upregulated in the OVA + IL-17A group. Therefore, it is interesting to speculate that TGF-β1 may also contribute to IL-17RA expression in the lung tissue, though hints from the literature which support this hypothesis are missing.

In summary, in this study we present a mouse model with enhanced neutrophilic lung inflammation. This enhanced inflammation is triggered by the specific conditions resulting from the combination of a TH2 response with the concomitant effect of IL-17A, leading to increased expression of subunits of the IL-17 receptor. The observed effects can be reproduced in vitro by stimulating human cell lines with IL-17A and IL-13, resulting in increased expression of IL-17 receptor subunits and increased production of IL-8. Therefore, it is interesting to speculate that in humans with TH2-mediated asthma, additional stimuli leading to IL-17A production also lead to increased neutrophil inflammation through increased IL-8 production due to increased expression of the IL-17 receptor.

## 5. Conclusions

In conclusion, the study showed that IL-17A, when combined with a TH2 response, leads to increased neutrophilic inflammation in the lungs. This effect was mediated through the enhanced expression of IL-17 receptor subunits, particularly IL-17RA. The findings suggest that in individuals with TH2-mediated asthma, additional stimuli triggering IL-17A production may contribute to heightened neutrophilic inflammation and IL-8 production, potentially exacerbating lung pathology. Further research is warranted to explore the mechanisms underlying these interactions and their significance in human asthma.

## Data Availability

All data is provided in the manuscript and supplement.

## Supplementary Materials

The following supporting information can be downloaded at: https://www.mdpi.com/article/10.3390/cells12131774/s1, Figure S1: Measurement of CXCL1 and total protein in BALF. (A) CXCL1 was measured in BALF supernatant by ELISA. (B) total protein in BALF supernatant was measured by a Bradford assay and quantified by preparing a standard curve with bovine serum albumin. Statistical significance is determined by comparing the OVA with OVA + IL17A group by one way ANOVA test ** *p* < 0.01, **** *p* < 0.0001. Figure S2: Alcianblue-PAS staining of goblet cells. Representative Alcianblue-PAS staining of paraffin sections of the lung tissue of mice from the different groups. Proximal and distal bronchioles are shown at different magnification (Br = bronchiole, v = vessel, arrows indicate goblet cells). Figure S3: Sirius red staining of bronchiolar collagen layer. Representative Sirius red staining of paraffin sections of the lung tissue of mice from the different groups. Proximal and distal bronchioles are shown at different magnification (Br = bronchiole, v = vessel). Figure S4: detection of α-smooth muscle actin (αSMA) by immunohistochemistry. Representative αSMA staining of paraffin sections of the lung tissue of mice from the different groups. Indirect immunofluorescence staining was performed by using a primary antibody directed against αSMA, followed by an AlexFluor488 coupled second antibody. In the negative control the primary antibody was omitted. Proximal and distal bronchioles are shown (Br = bronchiole, ve = vessel). Figure S5: Allergic inflammation and remodeling in the airways of mice in a long-term challenge. A: Treatment of Balb/c mice, Group1: unstimulated *n* = 4, Group 2: IL-17A application intranasally (i.n.) *n* = 6, Group 3: Ovalbumin (OVA)-asthma *n* = 6, Group 4: OVA-Asthma + IL-17A i.n. *n* = 6 B and C: cells in BAL cytospins, D: goblet cells in proximal airway epithelium, cells, per 100µm of Alician blue PAS stained lung sections, E: Thickness of the basal lamina in proximal airways measured after sirius-red staining, one datapoint reflects 25 measurements of 5 bronchioles from one mouse. Statistical differences were calculated using one-way ANOVA Kruskal-Wallis Test, for differences to control groups Dunn‘s post-test was used. * *p* < 0.05; ** *p* < 0.01; *** *p* < 0.001.
