# Supplementary material for: Interaction of Interleukin-17A with a Th2 Response in a Mouse Model of Allergic Airway Inflammation
Source: Cells. 2023 Jul 4;12(13):1774. doi: 10.3390/cells12131774 (PMC10340318; doi:10.3390/cells12131774)
Supplement: Supplementary file 1 [file cells-12-01774-s001.zip › cells-2482101-supplementary.pdf]

# Supplemental Material

# Interaction of Interleukin-17A with a Th2 Response in a Mouse Model of Allergic Airway Inflammation

**Karin Peters [1], Stefanie Ernst [2] and Marcus Peters [1,*]**

[1] Department of Molecular Immunology, Ruhr-University Bochum, D-44780 Bochum, Germany
[2] Department of Experimental Pneumology, Ruhr-University Bochum, D-44780 Bochum, Germany
* Correspondence: marcus.peters@rub.de

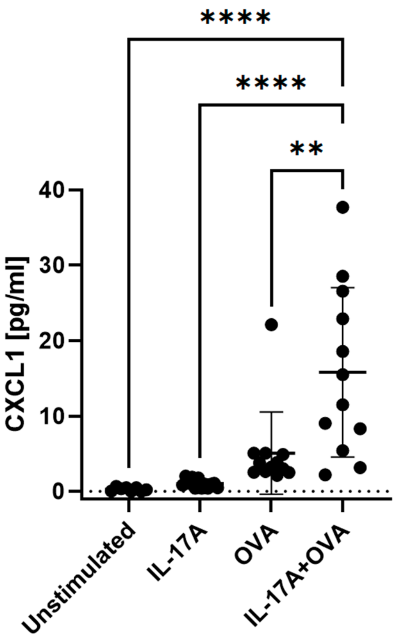


**A**

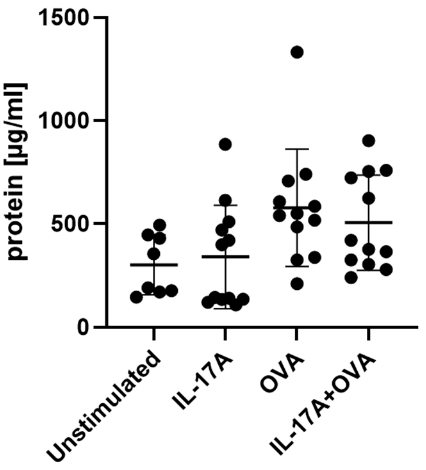


**B**

Supplemental Figure S1 : **Measurement of CXCL1 and total protein in BALF.** A) CXCL1 was measured in BALF supernatant by ELISA. B) total protein in BALF supernatant was measured by a Bradford assay and quantified by preparing a standard curve with bovine serum albumin. Statistical significance is determined by comparing the OVA with OVA+IL17A group by one way ANOVA test ** p<0,01, **** p<0,0001.

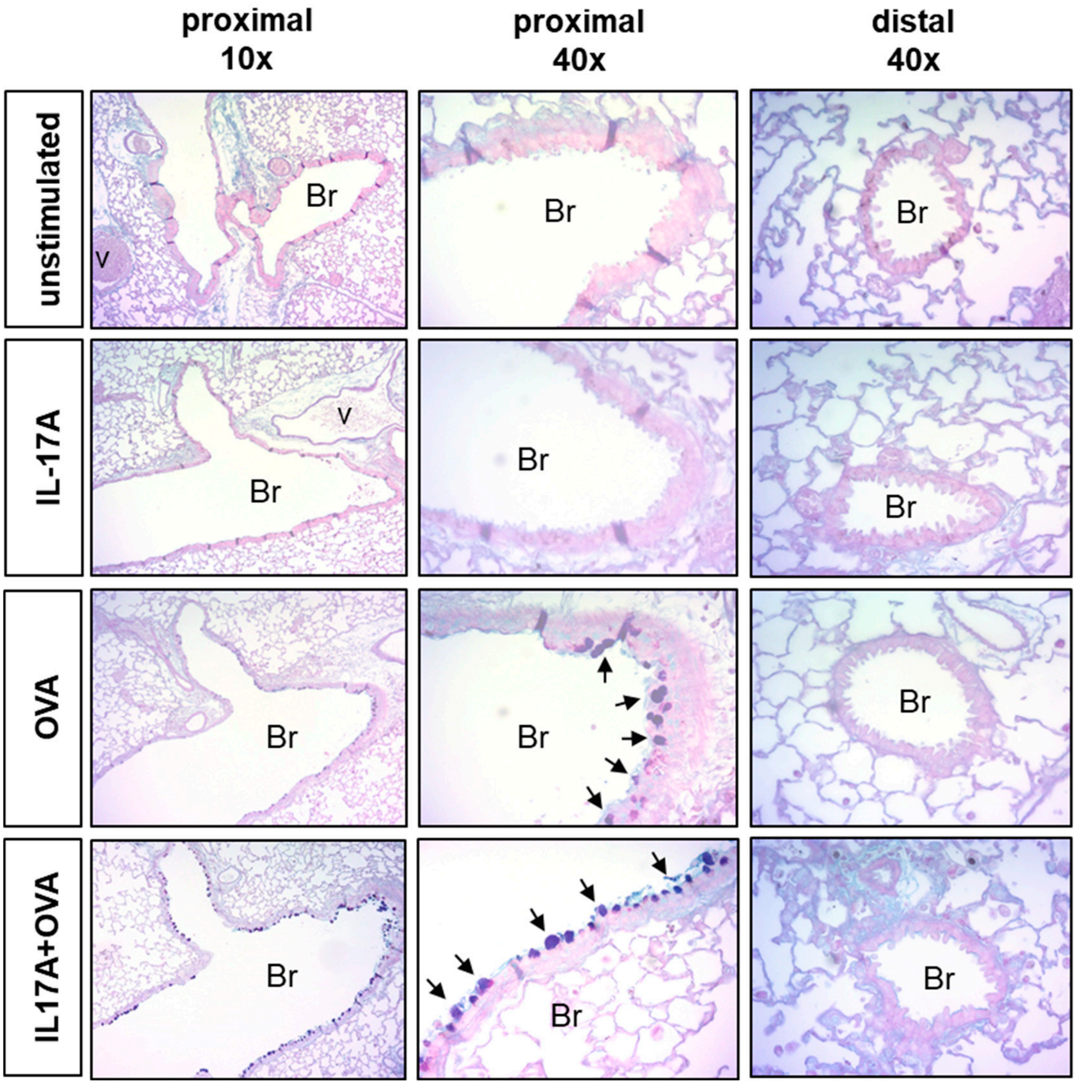


Supplemental Figure S2: **Alcianblue-PAS staining of goblet cells.** Representative Alcianblue-PAS staining of paraffin sections of the lung tissue of mice from the different groups. Proximal and distal bronchioles are shown at different magnification (Br=bronchiole, v=vessel, arrows indicate goblet cells)

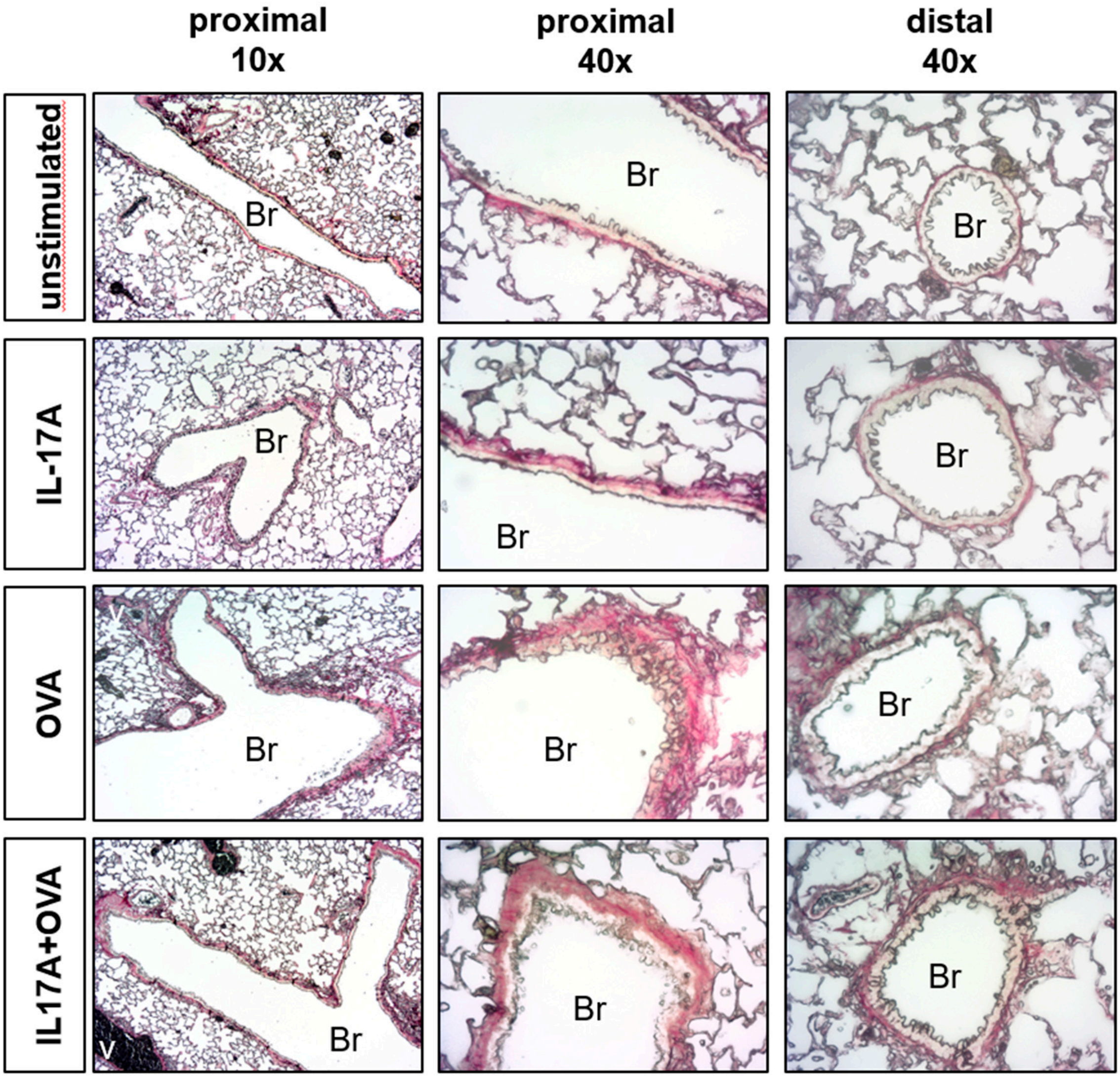


Supplemental Figure S3: **Sirius red staining of bronchiolar collagen layer.** Representative Sirius red staining of paraffin sections of the lung tissue of mice from the different groups. Proximal and distal bronchioles are shown at different magnification (Br=bronchiole, v=vessel)

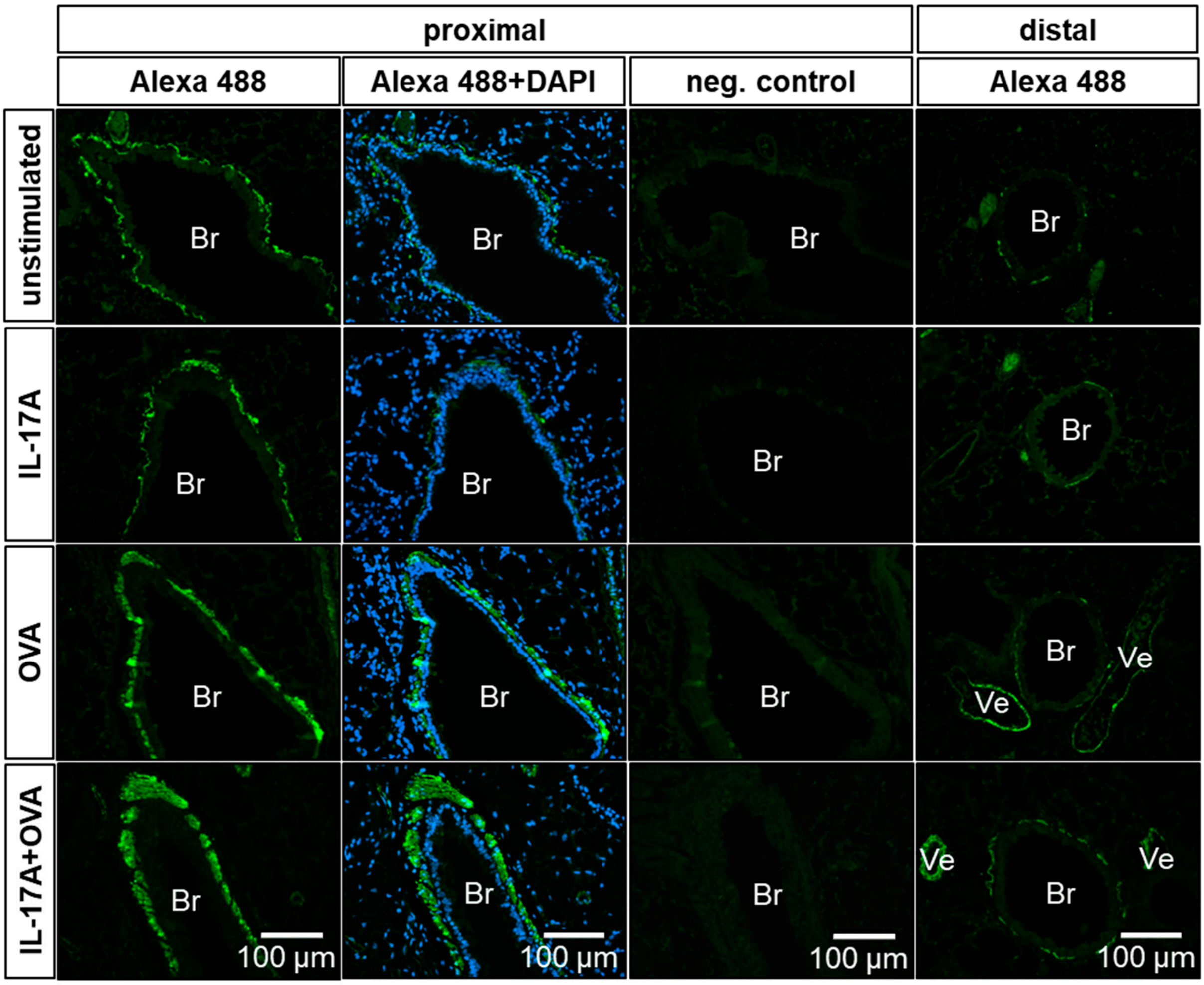


Supplemental Figure S4: **detection of α-smooth muscle actin (αSMA) by immunohistochemistry.** Representative αSMA staining of paraffin sections of the lung tissue of mice from the different groups. Indirect immunofluorescence staining was performed by using a primary antibody directed against αSMA, followed by an AlexFluor488 coupled second antibody. In the negative control the primary antibody was omitted. Proximal and distal bronchioles are shown (Br=bronchiole, ve=vessel)

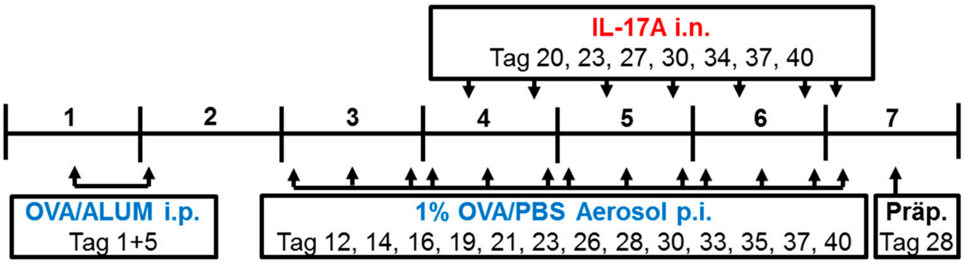

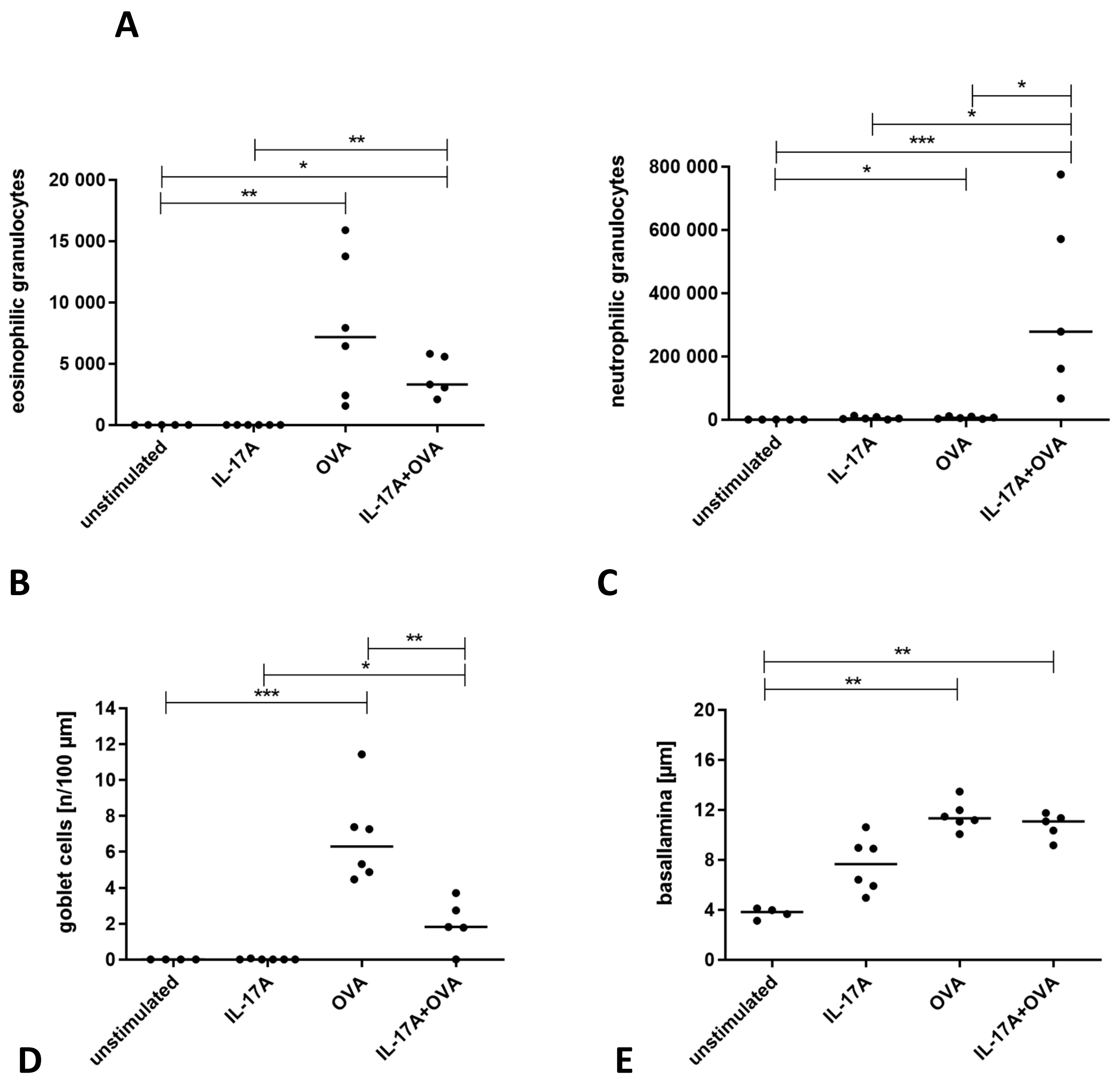


Supplemental Figure S5: **Allergic inflammation and remodeling in the airways of mice in a long-term challenge.** A: Treatment of Balb/c mice, Group1: unstimulated n=4, Group 2: IL-17A application intranasally (i.n.) n=6, Group 3: Ovalbumin (OVA)-asthma n=6, Group 4: OVA-Asthma + IL-17A i.n. n=6 B and C: cells in BAL cytospins, D : goblet cells in proximal airway epithelium, cells, per 100µm of Alician blue PAS stained lung sections, E: Thickness of the basal lamina in proximal airways measured after sirius-red staining, one datapoint reflects 25 measurements of 5 bronchioles from one mouse. Statistical differences were calculated using one-way ANOVA Kruskal-Wallis Test, for differences to control groups Dunn‘s post-test was used. *p<0,05; **p<0,01; ***p<0,001.
